# Characterization of Phytoestrogens in *Medicago sativa* L. and Grazing Beef Cattle

**DOI:** 10.3390/metabo11080550

**Published:** 2021-08-20

**Authors:** Jessica M. Wyse, Sajid Latif, Saliya Gurusinghe, Erica D. Berntsen, Leslie A. Weston, Cyril P. Stephen

**Affiliations:** 1 School of Agricultural, Environmental and Veterinary Sciences, Charles Sturt University, Wagga Wagga, NSW 2678, Australia; jwyse@csu.edu.au (J.M.W.); sgurusinghe@csu.edu.au (S.G.); cstephen@csu.edu.au (C.P.S.); 2 Graham Centre for Agricultural Innovation, Locked Bag 588, Wagga Wagga, NSW 2678, Australia; slatif@csu.edu.au; 3 Faculty of Science, National Life Sciences Hub, Building 289, Charles Sturt University, Wagga Wagga, NSW 2678, Australia; 4 Department of Agriculture, Falkland Islands Government, Stanley FIQQ 1ZZ, Falkland Islands; plantinteractions@csu.edu.au

**Keywords:** cattle, coumestans, lucerne, mass spectrometry, metabolic profiling, phytoestrogens, reproduction

## Abstract

Phytoestrogens are plant-produced bioactive secondary metabolites known to play an integral role in plant defense that frequently accumulate in times of stress and/or microbial infection. Phytoestrogens typically belong to two distinct chemical classes; flavonoids (isoflavones) and non-flavonoids (lignans and coumestans). Upon consumption by livestock, high concentrations of phytoestrogens can cause long-term disruption in reproduction due to structural similarities with mammalian estrogens and their tendency to bind estrogen receptors. Wide variation in phytoestrogen concentration has been reported in pasture legumes and corresponding silage or hay. Lucerne is a common perennial pasture legume in temperate climates, but information on phytoestrogen production or accumulation in grazing livestock is currently limited. Therefore, metabolic profiling using UHPLC-MS-QToF was performed to identify and quantitate key phytoestrogens in both fresh and dried lucerne fodder from replicated field or controlled glasshouse environments. Phytoestrogens were also profiled in the blood plasma of Angus cattle grazing field-grown lucerne. Results revealed that phytoestrogens varied quantitatively and qualitatively among selected lucerne cultivars grown under glasshouse conditions. Fresh lucerne samples contained higher concentrations of coumestans and other phytoestrogenic isoflavones than did dried samples for all cultivars profiled, with several exceeding desirable threshold levels for grazing cattle. Coumestans and isoflavones profiled in plasma of Angus heifers grazing lucerne increased significantly over a 21-day sampling period following experimental initiation. Currently, threshold concentrations for phytoestrogens in plasma are unreported. However, total phytoestrogen concentration exceeded 300 mg·kg^−1^ in fresh and 180 mg·kg^−1^ in dried samples of selected cultivars, suggesting that certain genotypes may upregulate phytoestrogen production, while others may prove suitable sources of fodder for grazing livestock.

## 1. Introduction

Lucerne (*Medicago sativa* L., also known as alfalfa) is a perennial, temperate pasture legume [1] that is widely utilized in mixed farming systems as a preferred source of cattle forage and/or fodder. In comparison to other pasture species, lucerne contains higher concentrations of protein and minerals, while its lower fiber content renders it beneficial for the provision of higher net energy for grazing livestock [2]. However, lucerne also produces numerous secondary plant metabolites, which may disrupt reproductive processes in livestock, cause bloating and result in reduced palatability. Currently, a wide range of cultivars are available in the Australian market, selected for differing climatic conditions, resistance to pests and/or pathogens [1], seasonality, and suitability for grazing, hay or silage production. Despite a wide selection of lucerne genotypes resulting from legume breeding and selection, lucerne grazed livestock may exhibit significant consequences associated with the production of bioactive secondary metabolites, including photocytotoxicity and infertility [3,4].

Legumes such as lucerne and clover (*Trifolium* spp.) produce significant quantities of non-steroidal secondary metabolites known as phytoestrogens [5]. Phytoestrogens are synthesized by various enzymatic pathways depending on the structural group [6] and are known to play an important role in plant defense against predation and herbivory [6], as well as plant growth and maintenance [7]. Plants tend to accumulate phytoestrogens during times of environmental stress and disease [8]. Due to structural similarities to mammalian estrogens (i.e., 17β-estradiol) and the tendency to bind estrogen receptors (ER) [9,10], significant phytoestrogen exposure in livestock can dramatically diminish reproductive efficiency [6]. Phytoestrogens became a focal area of research in the late 1980s, as estrogenic metabolites were found to have long-standing adverse impacts on both animal and human reproductive systems [6]. Livestock, particularly cattle and sheep, grazing legume pastures with higher phytoestrogen content have been shown to experience reproductive dysfunction ranging from lower conception rates and increased embryonic loss [11], to complete infertility [9].

**Figure 1:**
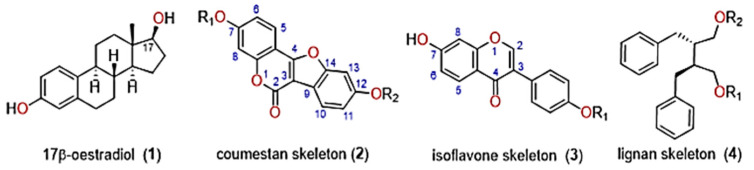
17β-estradiol (**1**) and the key classes of phytoestrogens, coumestans (**2**), isoflavones (**3**) and lignans (**4**).

Plant secondary metabolites that exhibit phytoestrogenic activity typically belong to two distinct structural groups; flavonoids (isoflavones) and non-flavonoids (lignans and coumestans) (Figure 1) [12]. Interestingly, the earliest studies on sheep infertility in Australia led to the identification of novel isoflavones in *T. subterraneum* L. (subterranean clover) [13]. Isoflavones predominate in legumes [14] and account for the greatest estrogenic activity in clover species such as *Trifolium pretense* L. (red clover) and subterranean clover, as previously reported in Australia [15,16,17]. Clover spp. and other legumes typically accumulate isoflavones as glycosides [18] that are generally further metabolized, resulting in complex classes of molecules, including pterocarpans and coumestans. Isoflavones play a critical role in plant defense against microbial infection [19] and the isoflavones daidzein, formononetin, genistein and biochanin A are key phytoestrogenic metabolites present in abundance in legume forages [12,15,16]. In grazing livestock, the bioavailability of isoflavones in blood plasma is influenced by the chemical structure of ingested isoflavones [18], as well as the age and gender of the animal. It has been observed that cattle are more sensitive than sheep to the influence of isoflavones [20] on estrous cycle; this is due to isoflavone inhibition of aromatase, which is essential for estrogen biosynthesis and development of ovarian follicles [20].

Figure 1 presents the lignan skeleton. Lignans are found in flaxseed, nuts, cereals, legumes, fruit and vegetables, while coumestans occur predominantly in legumes (Table S1). Coumestans exhibit greater potential for inhibition of estrous in contrast to isoflavones [21]. Coumestrol, 3′methoxycoumestrol and 4′methoxycoumestrol are key coumestans (Figure S1) exhibiting estrogenic activity [22] and accumulating at higher concentrations during fungal infections [23]. Of the phytoestrogens, coumestrol impacts reproductive function more than most other extrinsic phytoestrogens. However, its relative activity ranges between 100 to 3000 times less than intrinsic 17 β-estradiol and diethylstilbestrol, respectively [24]. Coumestrol generally occurs in abundance in certain rapidly growing legumes. The vegetable and legume species; leaf tissues in lucerne have been shown to contain the highest concentration of coumestans relative to other plant tissues [25].

Phytoestrogens typically exert their biological activity by (1) mimicking the action of endogenous estrogens; (2) acting as estrogen antagonists; (3) altering the pattern of synthesis and metabolism of endogenous hormones; or (4) modifying hormone receptor numbers. Exposure to a highly estrogenic diet during critical reproductive development periods, such as pre-puberty or gonadal maturity, may disrupt reproductive functions and fertility, in both humans and livestock (Table S2) [26]. In general, ingestion of phytoestrogens suppresses follicular development and estradiol synthesis, disrupts normal endocrine function, amplifies androgen levels, reduces estrogen levels and stimulates formation multi-oocytes [27]. Concentrations of coumestans (Table S2) in lucerne as low as 25 ppm can significantly decrease the ovulation rate of ewes [25,28].

Previous reports have provided sufficient evidence for the inhibitory impact of coumestans on bovine reproduction [29]. However, their presence in contemporary legume crops, experiencing moderate to low annual rainfall and extremes of temperature, both stressors that serve as potential triggers for the overexpression of phytoestrogens, has been unreported. Several anecdotal reports from veterinarians and practitioners in the Riverina region of NSW have described fertility problems in livestock that may be associated with higher concentrations of coumestans in lucerne fodder.

Currently, there is a significant gap in the literature related to phytoestrogen concentration in commercial lucerne cultivars and impacts on reproduction in cattle, sheep and horses grazing lucerne. Recent advances in analytical chemistry have enabled rapid and accurate profiling of secondary metabolites, including phytoestrogens, with enhanced sensitivity and detection at parts per billion (ppb) levels [30]. Therefore, the objectives of this research were specifically to, (a) further investigate phytoestrogen production in current commercially available cultivars of lucerne under both field and glasshouse conditions using metabolic profiling with sensitive LC/MS QToF instrumentation and, (b) characterize and quantify phytoestrogen levels in field-grazed lucerne and grazing beef cattle under field conditions.

## 2. Results

### 2.1. Metabolic Profiling of Selected Lucerne Cultivars

**Figure 2:**
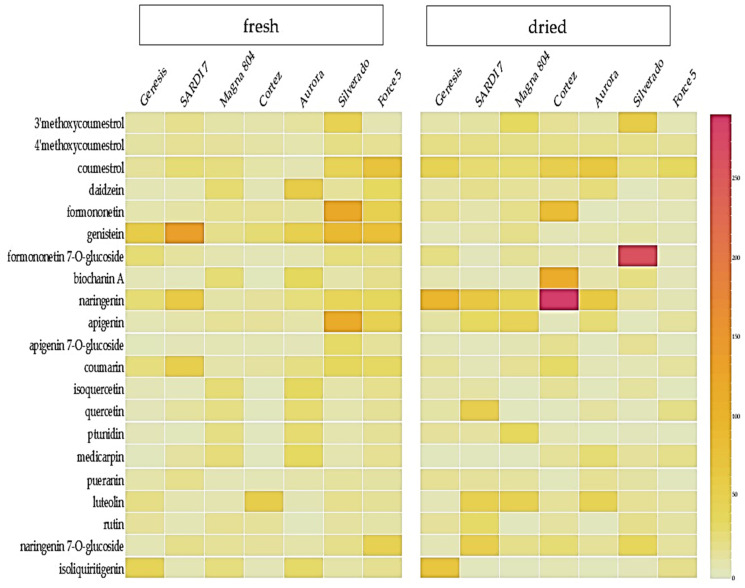
Heat map demonstrating the relative abundance of flavonoids in fresh and dried samples from seven lucerne cultivars produced under glasshouse conditions.

**Figure 3:**
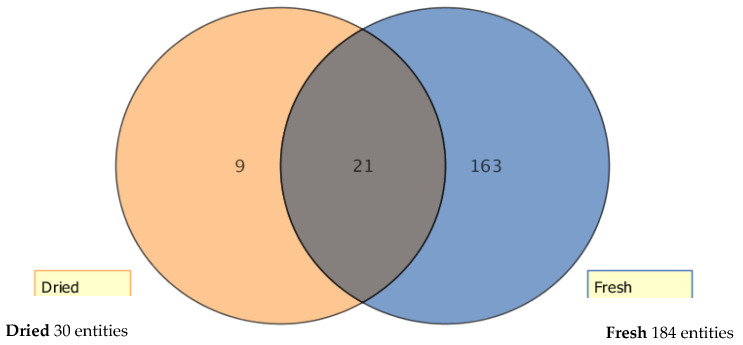
Venn diagram of flavonoids quantified in fresh and dried cultivars, and total flavonoids shared under both conditions.

Molecular features extracted using a non-targeted metabolic profiling approach were subjected to noise removal and normalization followed by targeted identification of key phytoestrogens in lucerne samples. A total of 1240 molecular features were profiled from extracts of either fresh or dried tissue samples. Further identification of molecular features of interest, phytoestrogens in particular, was performed by comparing their retention time (RT) and accurate mass with analytical standards, as well as a comparison with METLIN mass spectral database [31]. Phytoestrogens including, flavones, isoflavones, coumestans, and their respective glycosides, accounted for the majority of constituents in the fresh shoot samples of all lucerne cultivars presented in Figure 2. The metabolites and their respective glycosides identified and presented in Figure 2 are derivatives from the flavonol chalcone pathway associated with production of coumestrol, daidzein, formononetin and genistein. Interestingly, hierarchal clustering of genetically related cultivars was observed. For example, the cultivar Genesis is a progenitor of the more recently developed SARDI 7 cultivar (Figure S3), and both profile similarly and cluster together in hierarchal clustering. We observed that the concentration of certain flavonoids and other metabolites in lucerne foliar tissues differed between dried samples and fresh samples and was cultivar dependent (Figure 2 and Figure 3). Higher concentrations of specific flavonoids such as coumestrol, narigenin and luteolin in dried lucerne samples suggest their chemical stability during the harvesting and drying process experienced under field conditions as well. Genistein and coumarin were often present in notably higher concentrations in fresh tissues versus dried when cultivars were compared under glasshouse conditions (Figure 2).

Approximately 163 flavonoids were annotated in fresh foliar tissues of lucerne (Figure 3), with only 21 flavonoids found in both fresh and dried foliar treatments, while far fewer (9) were found only in the dried samples, presumably as a glycoside or methylated form of a precursor metabolite.

**Figure 4:**
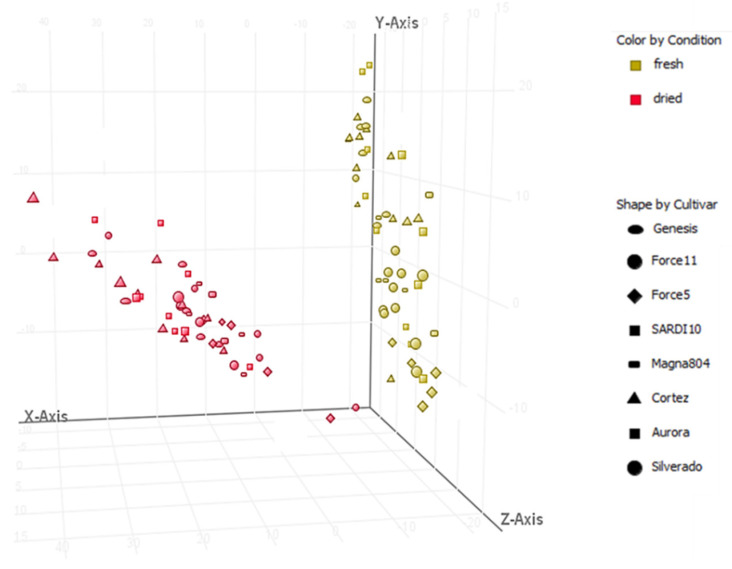
A visual representation of a 3D PCA (principal component analysis) of the molecular features characterized in shoot samples collected from seven cultivars of lucerne using UHPLC-QToF-MS in negative ion mode. Component 1 (X-Axis), 2 (Y-Axis) and 3 (Z-Axis) contributed to the separation by 48.63%, 10.82% and 14.05%, respectively.

The coumestans coumestrol, 3′methoxycoumestrol, 4′methoxycoumestrol, and isoflavones genistein, daidzein and formononetin were consistently detected in lucerne extracts prepared from aerial portions of lucerne plants maintained in glasshouse or field conditions. An unsupervised three-dimensional principal component analysis (3D PCA) demonstrated distinct clustering of spatially separated entities in fresh and dried samples (Figure 4). The clustering of such molecular features was observed based on the state of extracted plant tissue (fresh or dried) and potential relationships between the cultivars selected.

### 2.2. Quantification of Phytoestrogens in Lucerne Cultivars

Six key phytoestrogens, coumestrol, 3′methoxycoumestrol, 4′methoxycoumestrol, daidzein, formononetin and genistein, previously reported in legumes and other broadleaf pasture crops in the literature, were identified in all seven cultivars investigated (Table 1, Figure S1).

The concentration of coumestrol and 4′methoxycoumestrol differed significantly (*p* < 0.05) between fresh and dried samples, with dried samples containing higher concentrations on a mg·kg^−1^ dry matter basis (given that pre-determined moisture levels in fresh tissue allowed direct comparison on a dry weight basis). The concentration of genistein also varied significantly (*p* < 0.05) between fresh and dried samples, with fresh samples containing higher concentrations on a mg·kg^−1^ dry matter basis; however, significant differences between metabolite profiles for other constituents across cultivars were generally not observed. Coumestrol accumulated at higher concentrations in comparison to the coumestans 3′methoxycoumestrol and 4′methoxycoumestrol, while genistein accumulated at higher concentrations in contrast to the other isoflavones isolated in this study.

### 2.3. Quantification of Phytoestrogens in Field-Grown Genesis Lucerne

**Figure 5:**
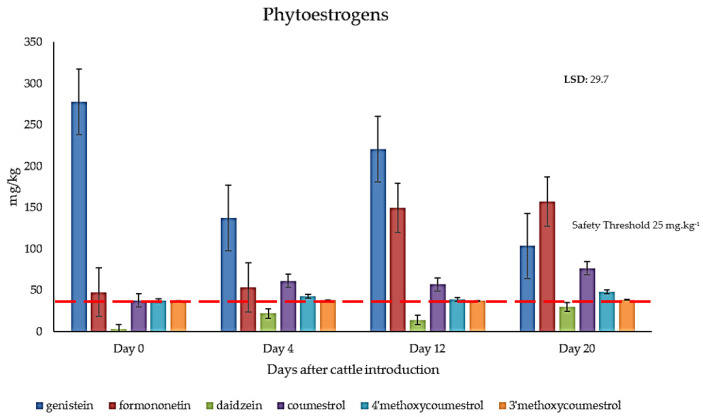
The average concentration of six phytoestrogens in mg·kg^−1^ in field-grown lucerne pastures cv. Genesis in Wagga Wagga NSW, following cattle introduction to the grazing trial on Day 0. The safe threshold for phytoestrogen is reported to be <25 mg·kg^−1^ of DM for livestock species investigated [29].

Coumestans and isoflavones were detected in all field-collected samples of lucerne cv. Genesis (Figure 5). A significant interaction (*p* < 0.05) was observed for the sampling date with respect to the production of coumestrol, 4′methoxycoumestrol, genistein and daidzein, but not 3′methoxycoumestrol or formononetin (*p* > 0.05). The concentration of coumestrol in cv. Genesis foliage was greater than other coumestans detected, while genistein was detected in the highest concentrations when compared to other isoflavones.

All six phytoestrogens quantified in this study over the four sample dates were noted at concentrations greater than the recommended safe threshold for livestock (<25 mg·kg^−1^ DM), with the exception of daidzein on Day 0 and Day 12. Remarkably, genistein concentrations were up to 5-fold higher in the field-grown Genesis lucerne, compared to glasshouse-grown lucerne, while coumestrol concentrations were up to 7 times higher in field-collected foliar samples.

### 2.4. Quantification of Phytoestrogens in Plasma Samples of Grazing Cattle

A significant difference (*p <* 0.05) for each of the phytoestrogens detected in cattle plasma was observed between treated (lucerne-fed) and control cattle, with the exception of genistein. A significant difference (*p <* 0.05) was also observed between control and lucerne grazed cattle over time (Table 2).

Interestingly, trace concentrations of coumestrol were detected in several heifers in the control group, likely due to the presence of small stands of coumestrol-producing red clover found intermittently in grazed control paddocks.

**Figure 6:**
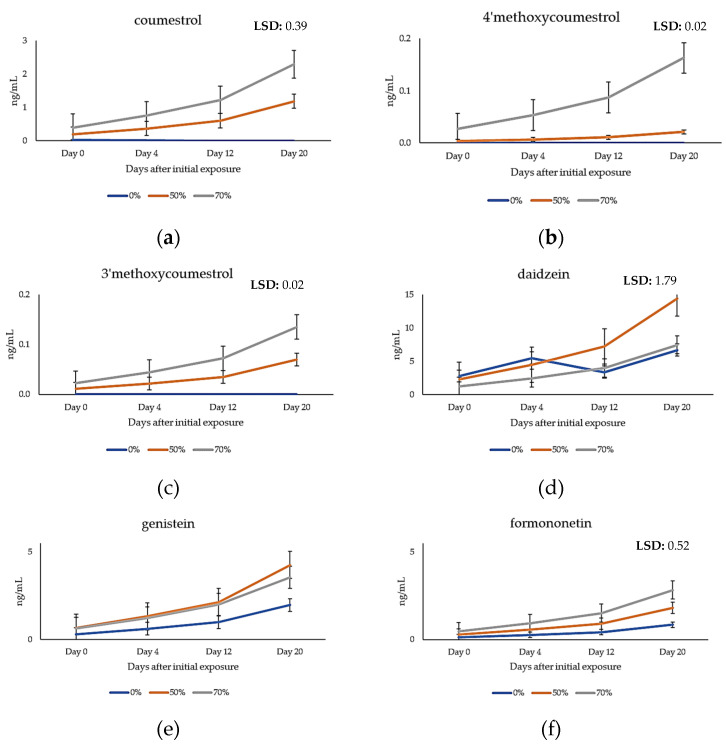
The average concentration of accumulated: (**a**) coumestrol; (**b**) 4′methoxycoumestrol; (**c**) 3′methoxycoumestrol; (**d**) daidzein; (**e**) genistein; and (**f**) formononetin; in the plasma (ng·mL^−1^) of the control (ryegrass) and two lucerne treatment groups (1. 50% lucerne stands; and 2. 70% lucerne stands) over four sampling times during a 21-day period.

The trends observed in the plasma concentration of phytoestrogens in lucerne grazed cattle (Figure 6) were similar to those observed in lucerne pasture samples and showed a marked increase in phytoestrogen concentration over time, with the exception of the isoflavone daidzein. For each of the sampling dates (day 0 through day 20), treatment groups exposed to 70% lucerne infestations in the paddock had higher concentrations of each of the phytoestrogens, and concentration increased over time in contrast to the treatment group exposed to 50% lucerne stands, which exhibited lower concentrations for each of the phytoestrogens, with the exception of daidzein (Figure 6d) and genistein (Figure 6e), where the 50% treatment group exhibited higher concentrations.

## 3. Discussion

### 3.1. Metabolic Profiling of Key Phytoestrogens in Lucerne Cultivars

Evaluation of lucerne grown under glasshouse conditions provided useful insights into the variation in biosynthesis and accumulation of phytoestrogen in selected lucerne cultivars grown to maturity. This experiment was performed in an attempt to alleviate the confounding effects of biotic and abiotic factors that may have typically influenced the expression of coumestans in the field, such as fungal and foliar pathogens, extreme temperatures and moisture stress [32]. Concentrations of coumestans in foliar tissues of lucerne varied significantly between cultivars under study, and we potentially attribute these variations to underlying cultivar genetics [3], suggesting that cultivars do clearly differ in their capacity to regulate the biosynthesis of coumestans. The majority of lucerne cultivars examined in this study originate from one the first Australian cultivars, Hunter River *cv.*, which was naturalized from the first French varieties of lucerne introduced to Australia in 1806. The cultivars evaluated in the glasshouse trial, Silverado, Genesis, SARDI 7 and Aurora, all share a closely related parental line (Figure S3) with Genesis, the predecessor to SARDI 7 and resulting genotypes.

Currently, a safe threshold for phytoestrogens of 25 mg·kg^−1^ is suggested for grazing livestock and is widely reported in the literature for potential prevention of reproductive impairment in both sheep and cattle [3]. However, foliar tissues of glasshouse grown SARDI7 and Silverado prepared as fresh extracts contained a higher concentration of coumestrol than the reported threshold value. Genetically related cultivars Genesis and SARDI 7 also contained concentrations of coumestrol at levels above the recommended safe threshold (25 mg·kg^−1^) in dried tissue extracts. In such cases, temporary infertility is likely to occur without displaying any outwardly evident signs and as such can only be detected through the quantification of coumestans in the diet or through plasma sampling of mammals [29]. Further clinical trials are therefore required to establish a clear association with higher concentrations of coumestans in lucerne cultivars and reproductive disruption. With respect to 3′-methoxycoumestrol, only cultivar Magna 804 contained above threshold levels in dried lucerne while Silverado contained estrotoxic levels both in the fresh and dried tissues, again suggesting a potential basis for variance in phytoestrogen production among genotypes.

Interestingly, 4′methoxycoumestrol concentrations above the recommended safe threshold were not noted. However, coumestrol levels in both lucerne hay and pasture were previously observed to be greater than 100 mg·kg^−1^ [3]. Coumestrol levels observed in the seven cultivars analyzed in this study did not exceed 100 mg·kg^−1^ in either fresh or dried samples. However, it is important to note that the total bioaccumulation of phytoestrogens (in both fresh and dried foliar samples) was much higher than that recorded for each of the individual metabolites, exceeding 300 mg·kg^−1^ in total in fresh samples and over 180 mg·kg^−1^ in dried samples. Our results suggest that while phytoestrogens may be produced above the reported threshold safety level in a controlled glasshouse environment, additional field experimentation is required for further verification in diverse lucerne genotypes.

### 3.2. Metabolic Profiling of Key Phytoestrogens in Genesis Lucerne and Cattle Plasma

No distinguishable pest or pathogen infestations were noted in field-grown lucerne. Thus, variations observed in the chemical profile of phytoestrogens could logically be attributed to numerous abiotic stimuli including, but not limited to, nutrient availability, temperature, and precipitation. Results of this grazing trial demonstrated that an established lucerne pasture (3 to 5 years of age) has the potential to result in in vivo coumestrol concentrations greater than the recommended threshold levels for cattle (25 mg·kg^−1^). The concentration of coumestrol exceeded this threshold level in cattle grazed on lucerne for each of the four sampling dates, with the greatest concentration recorded on day 20 (76.5 mg·kg^−1^), the final day of sampling in this trial. As the lucerne matured, concentrations of coumestrol and other phytoestrogens during the grazing trial generally increased *in planta* and were well above the recommended threshold in the lucerne, except for daidzein. The combined accumulation, however, of the six quantified phytoestrogens exceeded well over 500 mg·kg^−1^, and this coupled with other isoflavones, flavones and coumestans profiled, suggests that the cattle subjected to this grazing trial may well have experienced in vivo effects of phytoestrogens associated with accumulation in body fluids and tissues following grazing on an established lucerne pasture. Research is currently underway to evaluate clinical impacts of total phytoestrogens in cattle during and post-grazing. Our results also imply that ingestion of field-produced fresh lucerne may also disrupt reproductive function in grazing livestock than dried fodder, and further investigation is warranted.

### 3.3. Metabolism of Phytoestrogens

Metabolic profiling results indicated that coumestrol was present in lucerne in at least two methylated forms, 3′ and 4′methoxycoumestrol. Previously, 4′methoxycoumestrol was reported in higher concentrations than coumestrol in lucerne pastures [33]. In this study, 4′methoxycoumestrol was found up to be up to 2-fold higher in the lucerne treatments than 3′methoxycoumestrol, but in a 0.5:1 to 1:1 ratio with coumestrol. Such methylated metabolites are often de-methylated after ingestion by ruminants, reverting to coumestrol [29]; we noted that 3′ and 4′methoxycoumestrol were found in a 0.1:1 ratio to coumestrol in the plasma of cattle foraging on fresh forage containing 50% or 70% lucerne. However, the estrotoxic concentrations of coumestrol or 3′ and 4′methoxycoumestrol in the plasma of beef cattle grazing lucerne have not yet been reported. This is the first study to profile cattle plasma for both coumestrol and methylated coumestrols following grazing on lucerne-based pasture. Madej and Lundh [18] previously reported that the coumestrol concentration observed in the blood stream of dairy cattle was 0.1 ng·mL^−1^ as assessed by liquid chromatography. Coumestrol found in other ruminants and equine subjects ranged from 3.7–8.1 ng·mL^−1^ in ewes, 2–3.9 ng·mL^−1^ in goats and 0.24–0.3 ng·mL^−1^ in mares [3,9,33,34]. The concentration of ingested methylated coumestrols was previously reported in mare plasma and methoxycoumestrol ranged from 0.06–0.18 ng·mL^−1^ [3]. Unfortunately, assessment of methylated coumestrols can frequently be compromised by chemical changes including de-methylation after sampling, extraction methods utilized, or insensitivity and lack of precision in analytical instrumentation employed. Careful sample handling procedures and rapid extraction and processing of samples using UPLC/QToF mass spectrometry have generated results that indicate that coumestrol and 3′methoxycoumestrol occur in greater plasma concentrations than previously reported and were generally higher than those of 4′methoxycoumestrol. However, the factors impacting coumestan biosynthesis and their direct effects on livestock are largely not well understood. Therefore, additional research is warranted to improve our understanding of their relative potency in ruminants.

### 3.4. Biosynthesis of Phytoestrogens

The dominant isoflavone recovered in cattle plasma in this study was genistein, followed by formononetin and daidzein. Isoflavones were expressed at higher concentrations in the treatment group when compared to the control group, but no significant differences in isoflavones (daidzein, formononetin and genistein) were noted (*p* > 0.05) between treatment groups. This could potentially be due to the presence of isoflavones in both the ryegrass control as well as lucerne and small infestations (<5%) of white clover observed in several control replicates. Among isoflavones, genistein and daidzein were found in greatest concentrations in field-grown lucerne. Transformation of formononetin by demethylation to daidzein may also occur *in planta* [35]. This is supported by the fact that formononetin was present at greater concentrations in field-collected lucerne samples than those of daidzein. These results are consistent with a recent report evaluating isoflavone production in perennial legumes at flowering [36]. Specifically, genistein and daidzen were noted in higher concentrations in lucerne samples, with formononetin present at lower levels. This trend was also observed in results obtained in plasma samples collected in grazing heifers. It is possible that degradation of genistein to p-ethyl phenol (which is not estrogenic) may have occurred as previously reported, but we did not assess p-ethyl phenol levels [35]. This transformation is typically induced in ruminants after several days of access to feed rich in isoflavone content [35]. The microflora within the rumen can rapidly de-methylate the methylated coumestans (3′-methoxy and 4′-methoxycoumestrol), thereby increasing biological activity, resulting in enhanced estrogenicity of the pasture [37]. However, as these compounds are degraded, the estrogenicity of the pasture will also typically decrease [37]. The means of ingestion of lucerne fodder by grazing cattle may influence coumestan metabolism in the rumen; metabolism differed both qualitatively and quantitatively when phytoestrogens were administered in a pure form compared to ingestion in glycosidic forms in plant-based fodders [38].

## 4. Materials and Methods

### 4.1. Greenhouse Trial

Seven cultivars of lucerne (*Medicago sativa*) were selected for evaluation under glasshouse conditions and subsequent metabolic profiling for phytoestrogens including coumestan (coumestrol, 3′ and 4′ methoxycoumestrol) and isoflavones (daidzein, formononetin, genistein). Each of the cultivars varied in dormancy and winter activity rating (WAR), with cultivars exhibiting lower dormancy ratings being more productive in the summer growing season and cultivars with higher dormancy ratings being more productive in the winter growing season (Table 3).

A potting mix was prepared at a ratio of 3:1:1:0.25 (potting mix: sand: soil: water) using Osmocote^®^ Professional Premium Plus potting mix, fine sand and red kandosol obtained from the Charles Sturt University, Wagga Wagga Agricultural Farm. Thirty-five seeds from each cultivar were inoculated with Nodule NTM peat slurry. The seeds were sown at a uniform depth of 5 cm. This was replicated 16 times. The pots were arranged in a randomized complete block design (RCBD). The cultivars were maintained under glasshouse conditions (22 °C daytime temperature, 15 °C night-time temperature) for 8 weeks. Twelve of the replicates were harvested after 8 weeks, before the flowering stage of the plant.

### 4.2. Plant Tissue Extraction

Plant material was collected from each cultivar from 6 replicates followed by drying at 40 °C for 48 h immediately post-harvest to simulate haymaking conditions. Moisture content was determined for each cultivar. The remaining 6 replicates of each cultivar were harvested, and flash-frozen at −20 °C for storage until further extraction. Plant material (5 g) from both fresh and dried samples was thoroughly mixed with quartz sand (particle size: 0.3–0.9 m, Büchi, Switzerland) and placed into 10 mL extraction cells. The plant tissue was later extracted using a pressurized solvent extraction system (E-916 Büchi, Switzerland), using two consecutive cycles under the following conditions; solvent: 100% methanol (HPLC grade, Chem Supply, Port Adelaide, SA, Australia), temperature: 35 °C, pressure: 1400 psi. Samples were dried using a rotary evaporator (Multivapour P-6, Büchi, Switzerland) at 35 °C followed by reconstituting to 20 mL in methanol. Samples were filtered through 13 mm × 0.2 um Agilent Econofilter polytetrafluoroethylene (PTFE) before storage at −20 °C. Consideration of extraction methods was undertaken during preliminary experimentation, and methanol was selected as the preferred solvent to preserve the glycoside forms of the metabolites during extraction. It also provided greater yield of moderately polar to polar compounds extracted.

### 4.3. Cattle Grazing Trial

Fifteen non-pregnant Angus heifers were selected for a grazing trial at Charles Sturt University assessing coumestan and isoflavone concentrations in plasma of individuals over a grazing period of 21 days. Animal study was approved by the Animal Care and Ethics Committee, Charles Sturt University (Protocol No. A19039), Wagga Wagga, Australia. All heifers were maintained on a ryegrass pasture for 12 days, before the commencement of the grazing trial, and were also supplemented with oaten hay. Heifers had access to water *ad libitum*, and standard husbandry practices were implemented. Prior to the feeding trial, estrus was synchronized using a 10-day CIDR^®^ (Controlled Internal Drug Release) program. Briefly, an intravaginal progesterone device (Eazi-Breed Cattle CIDR^®^, Zoetis Pty, Rhodes NSW Australia) containing 1.38 g of progesterone per CIDR^®^ was inserted intravaginally and each heifer received 1 mL Bomerol^TM^ (1 mg·mL^−1^ Oestradiol Benzoate Injection, Bayer Australia Ltd., Pymble NSW Australia) as an intramuscular injection (containing 1 mg of oestradiol benzoate). Eight days after insertion of the CIDR^®^, each animal received an intramuscular injection of 2 mL Estrumate (containing 250 µg cloprostenol/mL, MSD Animal Health, MacQuarie NSW Australia). The CIDR^®^s were removed 10 days after insertion and cattle were examined and confirmed to be in the same stage of estrous cycle. On day 1 of the grazing trial, venous blood samples were collected from the coccygeal vein to obtain control values. The heifers were then organized into experimental groups (Control (0% lucerne), T1 (50% lucerne stands), and T2 (70% lucerne stands), with five heifers per group. The pasture establishment treatments were selected and assessed for the botanical composition of lucerne, via the method used by Virgona et al. [39] (Table 4). The control group had access to 3 ha of ryegrass, while each of the treatment groups had access to 3 ha of Genesis lucerne. Genesis is a winter active lucerne variety, that is high yielding for both grazing and haymaking under both dryland and irrigated conditions. It is also highly resistant to most major diseases of lucerne in Australia. As a cultivar that has been widely adopted across Australia, it was selected for the grazing trial to determine phytoestrogen accumulation in the plant and cattle plasma. As a preventative measure to reduce the risk of bloat from rapidly growing lucerne pasture, each animal received 25 mL of Vicchem Oral Bloat Drench^TM^, Coolaroo VIC Australia on day 1 of the trial. The bloat drench was also added to the water troughs at a ratio of 0.26:1 L (drench: water) on day 0.

The grazing trial was performed for 21 days, with five pasture plant and plasma time points sampled over the experiment and collected every fourth day. Pasture samples collected from the treatment and control paddocks were sampled using a one-meter quadrant, with five sub-samples collected from each quadrant (modified method from McIntyre [40]). Samples generated for each treatment were maintained in the paddock at 4 °C, and then stored at −20 °C until analysis by UHPLC-MS-QToF for coumestans and other relevant phytoestrogens (formononetin, genistein and daidzein).

### 4.4. Blood Sampling and Extractions

Venous blood samples were collected from the coccygeal vein to assay for key phytoestrogen concentrations for the duration of the 21-day estrous cycle. After collection of blood into BD Vacutainer^®^ Heparin tubes, samples were centrifuged for 10× *g* at 22 g, to collect plasma (approximately 5 mL) from each of the individual samples and stored at −20 °C until further processing for extraction of coumestans (coumestrol, 3′methoxy and 4′methoxycoumestrol) and isoflavones (genistein, daidzein and formononetin). The method used for processing and analyzing the plasma samples was modified from the procedure used by Ludwig et al. [41]. Plasma samples were defrosted at room temperature, vortexed and centrifuged for 10× *g* at 5 °C. The samples were then mixed with a 1 mL solution of 2% formic acid in acetonitrile, and centrifuged for 10× *g* at 5 °C. Supernatant from the samples was then extracted and dried completely using a laboratory evaporator (GeneVacEZ-2 Plus, Tegent Technologies Ltd., Borehamwood, UK) at 12 mbar for 2 h at 22 °C. Samples were then resuspended in 1 mL of methanol and ultrasonicated for 2 min. Aliquots (1 mL) of the supernatant were then filtered using an Agilent 13 mm × 0.2 μm PTFE filter (Econofilter, Agilent Technologies, Melbourne VIC, Australia).

### 4.5. Metabolic Profiling of Phytoestrogens

Metabolic profiling of both plasma and plant samples was performed using an Agilent 1290 Infinity UHPLC system coupled with an Agilent 6530 quadrupole time-of-flight (QToF) mass spectrometer (MS; Agilent Technologies, Santa Clara CA, USA) with Dual Jet Stream ionization source (Agilent Technologies, Santa Clara CA, USA). Full scan mass spectra were received over a range of an *m/z* range of 100–1700 Da at a rate of two spectra/second in negative ion mode. Chromatographic separation was carried out with the use of a reverse-phase C18 Poroshell column (2.1 × 100 mm, 2.7 μm particle size) (Agilent Technologies, Santa Clara, CA, USA) equipped with a C18 guard column (2.1 × 12.5 mm, 5 μm particle size) (Agilent Technologies, CA, USA) using a flow rate of 0.3 mL·min^−1^. The column was equilibrated for 40 min prior to analysis. Reverse-phase separation was obtained using a gradient of solvent A, [water (Milli-Q, TKA-GenPure) + 0.1% formic acid (LC-MS grade, LiChropur^®^, 98–100%, Sigma-Aldrich, St. Louis, MO, USA)] and solvent B [95% HPLC-grade acetonitrile (RCI Labscan, Bangkok, Thailand) + 0.1% formic acid]. The solvent gradient was as follows: 5–100% B over 0–17 min for C18 chromatography. The DAD monitored the absorbance across varying wavelengths from 210 nm to 635 nm. The injection capacity for each sample was 10 μL. Nitrogen was used as the drying gas at 250 °C at a flow rate of 9 L·min^−1^.

### 4.6. Data Analysis

A matrix of molecular features characterized by mass to charge ratio (*m/z*) and retention time (RT) (Table 5) was generated using MassHunter Workstation Qualitative software version B07.00, MassHunter Profinder (version B.08.00), Mass Profiler Professional (MPP version 14.5) and Personal Compound Database Library (PCDL) (version B 07.00, Agilent Technologies, Santa Clara CA, USA). Parameters were as follows: peak height ≥ 5000 counts, compound ion count threshold two or more ions, compound alignment tolerances were 0.00% + 0.15 min for RT and 20.00 mg·kg^−1^ + 2.00 mDa for mass using MassHunter Profinder. Identification was performed by comparing molecular entities with PCDL having accurate mass, RT and mass spectra generated from analytical standards where possible and references to published literature (Figure S2). All descriptive statistical analyses were performed using Statistix (ver. 10.0, 2013), standard deviation and standard error of means were calculated and reported where applicable.

## 5. Conclusions

Results revealed that phytoestrogens varied quantitatively and qualitatively among selected lucerne cultivars grown under glasshouse conditions. Fresh lucerne samples contained higher concentrations of coumestans and other phytoestrogenic isoflavones than did dried samples for all cultivars profiled, with several exceeding desirable threshold levels for grazing cattle. Coumestans and isoflavones profiled in plasma of Angus heifers grazing lucerne increased significantly over a 21-day sampling period following experimental initiation. Currently, threshold concentrations for phytoestrogens in plasma are unreported. However, total phytoestrogen concentration exceeded 300 mg·kg^−1^ in fresh and 180 mg·kg^−1^ in dried samples of selected cultivars, suggesting that certain genotypes may upregulate phytoestrogen production, while others may prove suitable sources of fodder for grazing livestock.

## Figures and Tables

**Table 1:** The concentrations of most abundant coumestans and isoflavones identified in the lucerne cultivars grown under glasshouse conditions expressed in mg·kg^−1^ of dry matter (DM). LSD values indicate significant differences among cultivars for each metabolite at *p* = 0.05.

Cultivar	Fresh						
	Coumestrol	3′Methoxycoumestrol	4′Methoxycoumestrol	Daidzein	Formononetin	Genistein	TOTAL
Genesis	11.5 ± 3.7	8.7 ± 4.8	9.8 ± 7.9	2.9 ± 2.5	5.5 ± 2.6	57.0 ± 21.0	95.4
SARDI 7	25.2 ± 3.7	16.8 ± 5.8	14.6 ± 2.2	3.6 ± 3.4	9.2 ± 3.4	137.0 ± 21.1	206.5
Magna 804	21.8 ± 2.7	8.1 ± 4.4	11.6 ± 3.5	27.1 ± 7.7	15.6 ± 4.7	17.8 ± 4.2	102.1
Cortez	7.8 ± 0.6	6.2 ± 2.0	8.6 ± 2.6	3.6 ± 3.6	14.4 ± 6.4	26.0 ± 9.3	66.5
Aurora	4.1 ± 1.6	10.7 ± 2.8	5.4 ± 2.5	56.9 ± 11.0	9.8 ± 3.5	51.3 ± 11.5	138.2
Silverado	43.0 ± 17.4	46.1 ± 5.4	19.4 ± 7.8	12.8 ± 3.2	121.2 ± 36.0	91.3 ± 25.9	333.8
Force 5	73.0 ± 32.7	4.1 ± 1.5	14.9 ± 3.2	32.8 ± 21.3	50.7 ± 36.0	79.5 ± 32.1	255
LSD value	17.5	5.1	12.5	15.6	29.4	38.4	55.3
	**Dried**						
Genesis	44.2 ± 13.4	7.0 ± 1.1	19.7 ± 10.0	9.2 ± 1.0	16.6 ± 6.1	2.4 ± 1.4	99.1
SARDI 7	26.7 ± 8.2	10.0 ± 0.0	19.3 ± 5.8	17.4 ± 5.0	6.1 ± 1.6	6.5 ± 1.2	86
Magna 804	27.8 ± 9.9	32.8 ± 1.7	18.7 ± 12.7	13.2 ± 1.2	17.8 ± 6.2	18.3 ± 10.7	128.8
Cortez	52.8 ± 26.1	14.3 ± 2.0	17.9 ± 13.5	10.1 ± 2.5	84.2 ± 26.1	2.6 ± 1.6	181.9
Aurora	65.9 ± 4.7	8.3 ± 2.8	18.5 ± 17.0	23.6 ± 14.0	0.2 ± 0.1	5.6 ± 2.4	122.1
Silverado	24.0 ± 24.0	56.1 ± 1.2	17.9 ± 1.3	0.5 ± 0.5	2.5 ± 1.1	8.3 ± 3.3	109.4
Force 5	33.3 ± 4.1	3.4 ± 0.4	13.6 ± 4.1	7.1 ± 3.1	2.1 ± 1.4	3.7 ± 3.3	82
LSD value	23.8	34.8	10.8	9.9	21.5	11.5	51.8

**Table 2:** The average concentration of the six phytoestrogens (ng·mL^−1^) in the plasma of the control (C *n* = 5) and treated (T *n* = 10) cattle over the 21-day grazing period. Significant differences (*p <* 0.05) were observed between the control and treatment groups. *p* values indicate significant differences among cultivars for each metabolite.

	Day 0		Day 4		Day 12		Day 20	
	C	T	C	T	C	T	C	T
genistein	0.312 ± 0.01	0.651 ± 0.05	0.613 ± 0.06	1.279 ± 0.14	0.996 ± 0.04	2.079 ± 1.10	1.971 ± 1.08	3.912 ± 1.98
formononetin	0.134 ± 0.01	0.379 ± 0.02	0.263 ± 0.04	0.745 ± 0.07	0.427 ± 0.08	1.210 ± 0.91	0.845 ± 0.08	2.316 ± 0.91
daidzein	2.796 ± 1.12	1.758 ± 1.02	5.497 ± 2.12	3.456 ± 1.24	3.383 ± 1.15	5.616 ± 2.58	6.693 ± 3.35	10.925 ± 3.98
coumestrol	TD	0.285 ± 0.01	TD	0.561 ± 0.04	ND	0.911 ± 0.18	ND	1.739 ± 1.08
4′methoxycoumestrol	ND	0.015 ± 0.01	ND	0.030 ± 0.01	ND	0.049 ± 0.01	ND	0.092 ± 0.08
3′methoxycoumestrol	ND	0.017 ± 0.01	ND	0.033 ± 0.01	ND	0.054 ± 0.02	ND	0.205 ± 0.04
*p*-value	0.003		0.003		0.009		0.007	

TD = Trace amounts detected. ND = Undetected.

**Table 3:** Lucerne cultivars utilized in the glasshouse experiment with respect to dormancy, winter activity rating (WAR) and presence of seed coat (nitrogen inoculation) pre-planting.

* Cultivar	Dormancy	WAR	Seed Coat
^1^ Force 5	5	Semi winter dormant	Absent
^2^ Aurora	6	Semi winter dormant	Absent
^3^ SARDI 7	7	Winter active	Present
^3^ Genesis	7	Winter active	Present
^4^ Magna 804	8	Highly winter active	Absent
^5^ Silverado	9	Highly winter active	Absent
^4^ Cortez	9	Highly winter active	Absent

* Denotes supplier. ^1^ Seed Force, ^2^ Smyth Seeds, ^3^ Heritage Seeds, ^4^ Valley Seeds, ^5^ Upper Murray Seeds.

**Table 4:** Botanical composition of lucerne treatments in grazing trial.

Treatment Number	Lucerne%	Other Species * %
Control	0	100
1	50	50
2	70	30

* Other pasture species present in the botanical composition included; annual ryegrass *(Lolium rigidum* G.), white clover (*Trifolium repens* L.), barley grass (*Hordeum leprinum* L.), small-flowered mallow (*Malva parviflora* L.), fumitory (*Fumaria officinalis* L.) and shepherd’s purse (*Capsella bursapastoris* L.).

**Table 5:** Phytoestrogens from pasture legume species were identified in this study by UHPLC-QTOF-MS in positive ionization mode.

Name	Molecular Formula	Molecular Mass	[M-H]	Basis for Identification ^a^	RT
**Isoflavones**					
daidzein	C_15_H_10_O_4_	254.24	253.0506	STD	8.11
formononetin	C_16_H_12_O_4_	268.26	267.0663	STD	8.87
genistein	C_15_H_10_O_5_	270.24	268.0455	STD	7.75
**Coumestans**					
coumestrol	C_15_H_8_O_5_	268.22	267.0299	STD	7.88
3′methoxycoumestrol	C_16_H_10_O_6_	298.25	297.0405	AM	10.55
4′methoxycoumestrol	C_16_H_10_O_5_	282.25	281.0579	AM	8.77

^a^ Basis for identification codes: AM–match to accurate mass/molecular formula; STD–match to accurate mass and retention time of analytical standards.

## Data Availability

All data is stored in archived datasets as per the guidelines of Charles Sturt University and associated funding bodies.

## Supplementary Materials

The following are available online at https://www.mdpi.com/article/10.3390/metabo11080550/s1, Figure S1: Structure of common coumestan and isoflavones, Figure S2: Chromatogram for the determination of genistein, coumestrol, 4′methoxycoumestrol, fromononetin, daidzein and 3′methoxycoumestrol, Figure S3: Chromatogram for the determination of genistein, coumestrol, 4′methoxycoumestrol, fromononetin, daidzein and 3′methoxycoumestrol, Table S1: Notable coumestans also isolated from the Leguminosae family, Table S2: Summary of the reproductive effects induced from pasture species, and exhibited in livestock species.
