# Supplementary material for: Characterization of Phytoestrogens in Medicago sativa L. and Grazing Beef Cattle
Source: Metabolites. 2021 Aug 20;11(8):550. doi: 10.3390/metabo11080550 (PMC8398016; doi:10.3390/metabo11080550)
Supplement: Supplementary file 1 [file metabolites-11-00550-s001.zip › metabolites-1288062-SI-done.pdf]

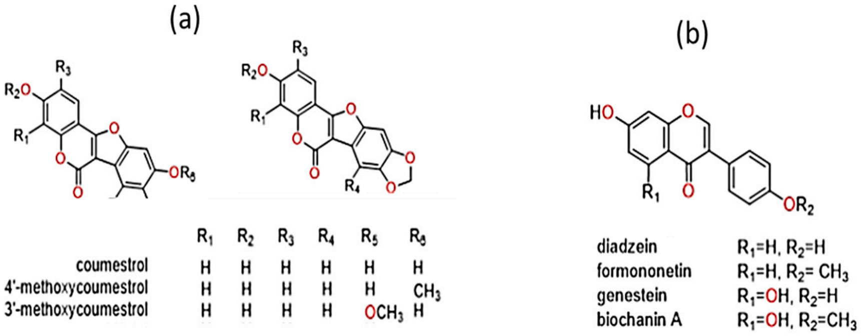

Figure S1. Structures of common (**a**) coumestans (coumestrol, 4'-methoxycoumestrol and 3'-methoxycoumestrol) and (**b**) isoflavones (daidzein, formononetin, genistein and biochanin A).

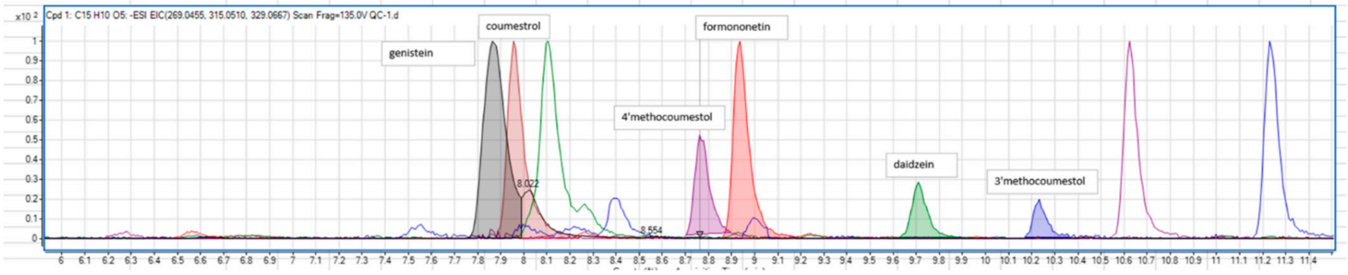

Figure S2. Layered chromatogram for the determination of genistein, coumestrol, 4’methoxycoumestrol, fromononetin, daidzein and 3’methoxycoumestrol in lucerne.

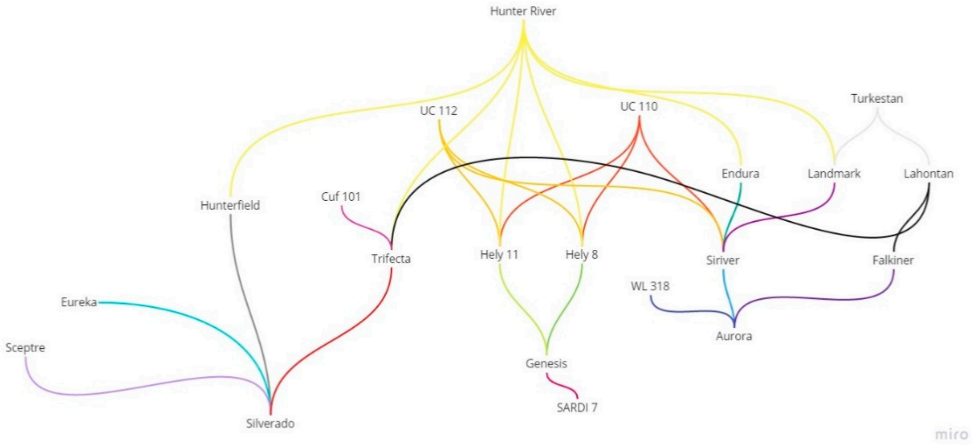

Figure S3. Genetic map of cultivars Silverado, Genesis, SARDI 7 & Aurora used in the greenhouse trial, and the cultivars used as a precursor to these.

Table S1. Notable coumestans also isolated from members of the *Leguminosae* family.

Coumestan	Plant Species	Molecular formula	*Molecular Mass*
coumestrol	*Medicago sativa*	$C_{15}H_8O_5$	268.22
3’methoxycoumestrol	*Medicago sativa*	$C_{16}H_{10}O_6$	298.25
4’methoxycoumestrol	*Medicago sativa*	$C_{16}H_{10}O_5$	282.25
trifoliol	*Trifolium repens* L.	$C_{16}H_{10}O_6$	298.25
repensol	*Trifolium repens* L.	$C_{15}H_8O_6$	284.22

medicagol	*Medicago sativa*	$C_{16}H_8O_6$	296.23
lucernol	*Medicago sativa*	$C_{15}H_8O_6$	284.22
sativol	*Medicago sativa*	$C_{16}H_{10}O_6$	298.25
7-hydroxy-11,12-dimethoxycou-mestan	*Medicago sativa*	$C_{17}H_{12}O_6$	312.27

Table S2. Summary of the reproductive effects induced from pasture species, and exhibited in livestock species.

Livestock Species	Reproductive Effects	Pasture Species	Reference
Bos taurus *(Bovine)*	Abortion	Red clover	
	Anestrus	Lucerne	
	Cystic ovaries	Lucerne	[9]
	Endometritis	Lucerne	
	Increased cervical mucus	Lucerne	
	Temporary Infertility	Lucerne	
	Inhibition of progesterone secretion	Red clover	
	Nymphomania	Red clover	
	Repeated, irregular, or false estrus	Lucerne	
	Swollen vulvae	Lucerne	[9]
	Uterine enlargement	Lucerne	
Ovis aries *(Ovine)*	Abortion		
	Dystocia	Subterranean clover	
	Hydrops uteri		[11]
	Temporary & complete infertility	Subterranean clover	
	Ovarian and endometrial cysts	Lucerne	
	Prolapse of uterus	Lucerne	
	Pyometron		
	Reduced ovulation, conception, and lambing rates	Lucerne	
	Severe metritis		[9]
Equus caballus *(Equine)*	Uterine hyper-oedema	Lucerne	[9]
	Persistent anovulatory follicles	Lucerne	
	Temporary in fertility	Lucerne	

Table S3. Standards utilised for the identification of flavonoids in the lucerne samples for comparison of accurate mass, retention time and key molecular features including mass spectrausing Personal Compound Database Library (PCDL) software (Agilent, USA).

Serial #	Flavonoids	Formulae	Mass	Retention Time
1	biochanin A	$C_{16}H_{12}O_5$	284.27	13.111
2	isoliquiritigenin	$C_{15}H_{12}O_4$	256.25	11.588
3	luteolin	$C_{15}H_{10}O_6$	286.24	10.021
4	naringenin	$C_{15}H_{12}O_5$	272.26	10.813
5	naringenin-7-O-glucoside (prunin)	$C_{21}H_{22}O_{10}$	434.4	8.726
6	puerarin	$C_{21}H_{20}O_9$	416.38	6.815
7	rutin	$C_{27}H_{30}O_{16}$	610.521	7.958
8	apigenin	$C_{15}H_{10}O_6$	270.24	10.862
9	quercetin	$C_{15}H_{10}O_7$	302.238	10.036
10	apigenin 7-O-glycoside	$C_{21}H_{20}O_{10}$	432.414	10.14
11	coumarin	$C_9H_6O_2$	146.1427	12.87
